# Formation of polysulfides as a smart strategy to selectively detect H_2_S in a Bi(iii)-based MOF material†

**DOI:** 10.1039/d4sc07144a

**Published:** 2025-02-19

**Authors:** Valeria B. López-Cervantes, Juan L. Obeso, J. Gabriel Flores, Aída Gutiérrez-Alejandre, Raul A. Marquez, José Antonio de los Reyes, Catalina V. Flores, N. S. Portillo-Vélez, Pablo Marín-Rosas, Christian A. Celaya, Eduardo González-Zamora, Diego Solis-Ibarra, Ricardo A. Peralta, Ilich A. Ibarra

**Affiliations:** a Laboratorio de Fisicoquímica y Reactividad de Superficies (LaFReS), Instituto de Investigaciones en Materiales, Universidad Nacional Autónoma de México Circuito Exterior s/n, CU, Coyoacán 04510 Ciudad de México Mexico argel@unam.mx; b Instituto Politécnico Nacional, CICATA U. Legaria, Laboratorio Nacional de Ciencia, Tecnología y Gestión Integrada del Agua (LNAgua) Legaria 694, Irrigación 11500 CDMX Mexico; c Área de Química Aplicada, Departamento de Ciencias Básicas, Universidad Autónoma Metropolitana-Azcapotzalco 02200 Ciudad de México Mexico; d Departamento de Ingeniería de Procesos e Hidráulica, División de Ciencias Básicas e Ingeniería, Universidad Autónoma Metropolitana-Iztapalapa 09340 Ciudad de México Mexico; e UNICAT, Departamento de Ingeniería Química, Facultad de Química, Universidad Nacional Autónoma de México 04510 Ciudad de México Mexico; f Department of Chemistry, The University of Texas at Austin Austin Texas 78712 USA; g Departamento de Química, División de Ciencias Básicas e Ingeniería. Universidad Autónoma Metropolitana (UAM-I) 09340 Mexico rperalta@izt.uam.mx; h Centro de Nanociencias y Nanotecnología, Universidad Nacional Autónoma de México Km 107 Carretera Tijuana-Ensenada Ensenada, B.C. C.P. 22800 Mexico; i Departamento de Química, Universidad Autónoma Metropolitana-Iztapalapa Avenida San Rafael Atlixco 186, Leyes de Reforma 1ra Sección, Iztapalapa Ciudad de México 09310 Mexico

## Abstract

SU-101 was demonstrated to be an effective and efficient detector for H_2_S, due to the facile generation of polysulfides, with a remarkable H_2_S selectivity. Raman and XPS analyses confirmed the formation of S_*n*_^2−^ and S_4_^2−^ polysulfide species after the H_2_S adsorption (at 0.05 bar, 0.1 bar and 1 bar), without compromising the structural integrity of SU-101. The detection mechanism involves rigidification of the structure by the formation of the polysulfides and blockage of the ligand–metal charge transfer (LMCT) process, which increased the radiative emission. Additionally, theoretical simulations were carried out in order to demonstrate that the interaction of the polysulfide molecules inside the pores of SU-101 is energetically stable. Remarkably, the limit of H_2_S detection (LOD) was calculated to be as low as approximately 22 ppm. Finally, SU-101 is nominated as a promising candidate for implementing toxic waste valorisation (*i.e.*, capture of toxic H_2_S) toward relevant applications in accurate molecular sensing.

## Abstract 2

Changes in the HOMO–LUMO energy of pristine and H_2_S-saturated SU-101 material and the Jablonski diagram explaining the changes in the fluorescence of the system.

## Graphical abstract

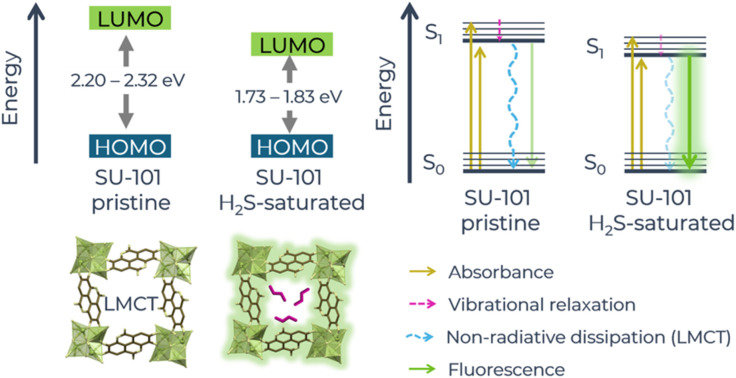

## Introduction

Hydrogen sulphide (H_2_S) is a toxic chemical in natural gas and biogas commonly emitted by different chemical industries. The desulphurization process of diverse gas streams (*e.g.*, oil refineries) is the primary source of H_2_S emissions.^1^ H_2_S is a colourless gas that is extremely corrosive, flammable, and lethal to humans.^2^ H_2_S is classified as a dangerous and major air pollutant, mostly associated with acid rain and severe nervous system illnesses.^3^

Thus, the efficient detection of H_2_S is desired for a cleaner environment and for human health protection. The development of methodologies for selective monitoring of H_2_S is necessary. Different technologies have been reported for H_2_S detection, such as colorimetry,^4^ electrochemical,^5^ and gas chromatography.^6^ However, implementing these methodologies requires a high measurement time and high complexity devices, which leads to a decrease in the possibility of practical applications.^7^ However, fluorescence techniques are much simpler alternatives for promising and potential H_2_S detection since the measurement method is reliable, user-friendly, convenient, and fast.^8^

Furthermore, different materials and strategies have been implemented for fluorescence detection of H_2_S. Carbon-based materials have been used to detect H_2_S in aqueous conditions and live cells.^9^ Silver nanoparticles were applied for H_2_S sensing in water.^10^ An alumina–graphene oxide nanocomposite was used for H_2_S detection in lake water.^11^ However, most of these materials are classified as free organic–inorganic molecules, which could exhibit fluorescence issues due to their molecular motion.^12^ Also, H_2_S detection is based on small molecules as fluorescent probes, such as inorganic sulphide salts (NaHS and Na_2_S). The detection is based on a chemical reaction between the material and the molecular probe *via* a nucleophilic attack, reduction, and precipitation to generate a fluorescence change.^13^ The implementation of robust materials for the detection of H_2_S molecules, by relatively strong interactions between H_2_S and the pore walls of the materials is highly desirable.

In this context, a comparatively novel class of highly crystalline and porous materials known as metal–organic frameworks (MOFs) has been recently explored for molecule detection.^14^ Based on some recent research in H_2_S adsorption,^15^ the high chemical stability, and the apparent advantages by only using small quantities,^16^ MOF materials are promising candidates for H_2_S detection. For example, fluorescence detection using MOF materials has been studied for detecting water pollutants,^17^ explosives^18^ and aromatic compounds.^19^ Specifically, MOF materials are also applied to detect H_2_S in the gas phase, for example, a Zr-MOF was used as a fluorescence turn-on detector for H_2_S.^20^ However, the fluoresce detection in the gas phase or the H_2_S quantification is poorly studied using MOF materials.

**Fig. 1:**
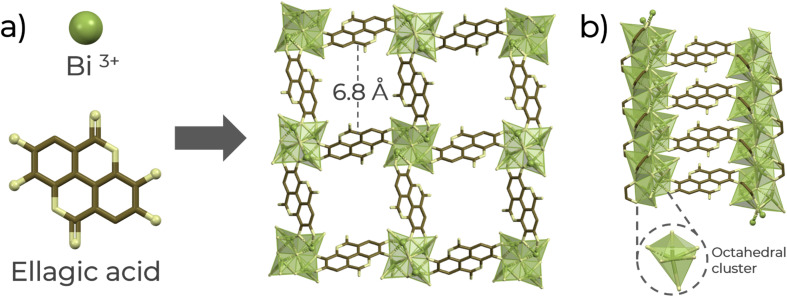
(a) Schematic of the formation of the SU-101 structure along the channel *a*-axis and (b) metal cluster and linker arrangement along the *a*-axis. Atoms label: green: Bi^3+^ octahedra, brown: carbon, and yellow: oxygen.

Thus, a chemically stable Bi(iii)-based MOF material denoted as SU-101 (ref. 21) (microporous bismuth ellagate framework (Bi_2_O(H_2_O)_2_(C_14_H_2_O_8_)·*n*H_2_O), C_14_H_2_O_8_ = ellagate) was selected to investigate qualitative H_2_S detection in the gas phase and quantitative sensing using a solution of H_2_S in dry THF. SU-101 shows each Bi(iii) metal centre is six coordinated, establishing three bonds with coordinating phenolates, two μ_4_-oxygens, and a terminally coordinated water molecule (Fig. 1), which can be removed after an activation process.^21^ Previously, SU-101 was reported for H_2_S adsorption, where it was demonstrated that the H_2_S adsorption generates spontaneous conversion into polysulfides.^21,22^ Then, taking advantage of such specific chemical transformation, the detection of H_2_S with SU-101 was investigated.

## Experimental section

### Synthesis of SU-101

SU-101 was synthesised following a previously reported procedure.^21^ 0.015 g of ellagic acid and 0.038 g of bismuth(iii) acetate were dissolved in 30 mL of a water and acetic acid mixture (6 vol% acetic acid). First, the solution was stirred vigorously at room temperature for 48 h. Later, the recovered powder was washed three times with water and ethanol. After that, the powder was dried overnight at 60 °C. Subsequently, the material was activated at 150 °C and under vacuum to evacuate any molecule trapped within the pores of SU-101.

### Instruments

Detailed information on the instrumental techniques is available in Section S1.

### Fluorescence experiments

Emission spectra were taken in an Edinburgh Instrument FS5 fluorimeter using a continuous wave 150 W ozone-free xenon arc lamp at room temperature, coupled with an SC-10 solid-state sample holder. Measurements were carried out using an excitation wavelength of 370 nm, with a 395 nm long-pass filter on the detector side to remove any remaining light from the excitation source. The measurements were collected with a step size of 1 nm and a dwell time of 0.2 s. The excitation bandwidth was set at 5 nm, and the emission bandwidth for the detector was set at 1 nm. Decay time spectra were measured by exciting the sample with a picosecond pulsed light emitting diode EPL-375 with an excitation wavelength of 374.2 nm and a pulse width of 52.9 ps at an emission wavelength of 460 nm. The samples were packed into quartz sample holders and positioned into the instrument for solid-state measurements. Both the activated and saturated samples were packed right after being taken out of the activation and saturation processes. A suspension of SU-101 in THF (1 mg mL^−1^) was prepared for the solution measurements. Also, solutions of H_2_S in THF at different concentrations (10–100 mM) were prepared. In this case, 2.5 mL of the SU-101 suspension and 0.5 mL of the various concentrations of H_2_S were mixed and measured.

### Computational methods

#### DFT calculations

To investigate the electronic structure properties of the SU-101 system, we performed density functional theory (DFT) calculations using SIESTA 4.1 (Spanish Initiative for Electronic Simulations with Thousands of Atoms) *ab initio* code.^23^ The geometries were optimised with the generalised gradient approximation (GGA), employing the Perdew–Burke–Ernzerhof (PBE) exchange–correlation functional to describe the electronic properties.^24^ Troullier–Martins pseudopotentials were applied,^25^ along with a single-ζ basis set, and an energy shift of 150 meV. The calculations adhered to stringent convergence criteria: 1 × 10^−4^ for total energy and electron density, and Hellmann–Feynman forces were converged to less than 0.05 eV Å^−1^ during structure optimization. An electronic temperature of 298 K was set for orbital occupation calculations using the Fermi–Dirac function. The Brillouin zone was sampled with a Monkhorst–Pack grid of 2 × 2 × 3 *k*-point for the modelled supercell. For optical properties, the imaginary part of the dielectric function was calculated using SIESTA code, which incorporates first-order dependent perturbation theory.^26^

## Results and discussion

### Characterization of SU-101

Powder X-ray diffraction (PXRD) confirmed the phase purity of SU-101 (Fig. S1†). At 2*θ*, peaks at 6.7, 9.6, 13.4, and 26.7° can be observed, which are in good agreement with the pure crystal structure.^21^ Fourier transform infrared (FT-IR) spectroscopy shows specific bands for the functional groups of SU-101 (Fig. S2†). The bands at 1703 and 1056 cm^−1^ are related to C=O stretching and O–H in-plane deformation from the organic linker.^21^ The band at 1367 cm^−1^ confirms the chemical coordination between the ellagic acid and the Bi^3+^ sites. The characteristic band of phenolate stretching vibration in the ellagate anion at 3411 cm^−1^ is observed. The band at 1465 cm^−1^ is related to the aromatic ring vibrations.^21^ The TGA profile of SU-101 (Fig. S3†) shows moderate thermal stability up to 280 °C and indicates that the guest solvent molecules can be removed at 150 °C after activation. SEM images (Fig. S4†) show the morphology characteristics of SU-101, displaying a homogeneous columnar crystal.

### H_2_S adsorption performance

Prior to the detection performance for SU-101, an activated sample was evaluated for H_2_S uptake. A breakthrough H_2_S experiment (4.3 vol% H_2_S with 95.7 vol% of N_2_) led to a gas uptake of 15.64 mmol g^−1^, *i.e.*, 531 mg g^−1^ (Fig. S6†). This H_2_S capture value is in good correlation with the previous value reported (15.95 mmol g^−1^, *i.e.*, 542 mg g^−1^),^21^ and on a second H_2_S adsorption cycle, the initial capacity was lost (0.1 mmol g^−1^, Fig. S7†), which confirms the formation of polysulfides inside the pores of SU-101, as we previously observed.^21^ The chemical stability was confirmed by PXRD (Fig. S8†); moreover, the effectiveness of SU-101 in detecting H_2_S due to the chemical transformation of H_2_S into polysulfides was evaluated. As a proof of concept, the conversion of H_2_S (to polysulfides) as a function of time was analysed using gas chromatography (Table S1†). The conversion of H_2_S was observed from 0.06 mmol g^−1^ at 120 s to 0.73 mmol g^−1^ at 670 s. It is worth mentioning that once SU-101 is exposed to H_2_S, due to the chemical transformation to polysulfides, the sample cannot be re-used (re-activated).

### Fluorescence H_2_S detection

**Fig. 2:**
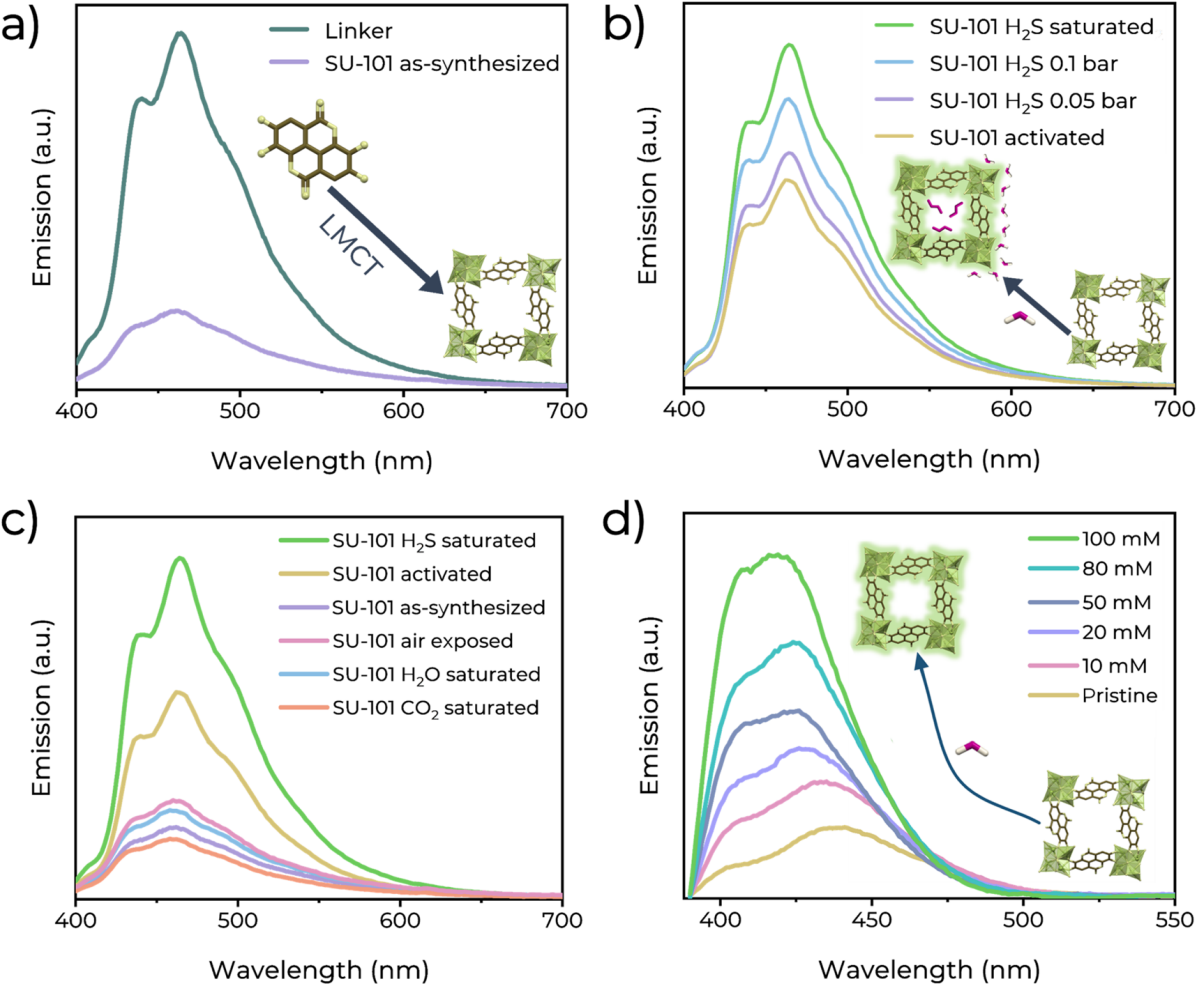
(a) Solid-state emission spectra of the ellagic acid linker (brown) and as-synthesized SU-101 (yellow). (b) Comparison of the solid-state emission spectra of activated SU-101 and SU-101 exposed to different H_2_S gas pressures. (c) Comparison of the solid-state emission spectra of SU-101 exposed to air, water vapour and different gases. (d) Fluorescence emission spectra of dispersed SU-101 in H_2_S solutions in THF.

**Fig. 3:**
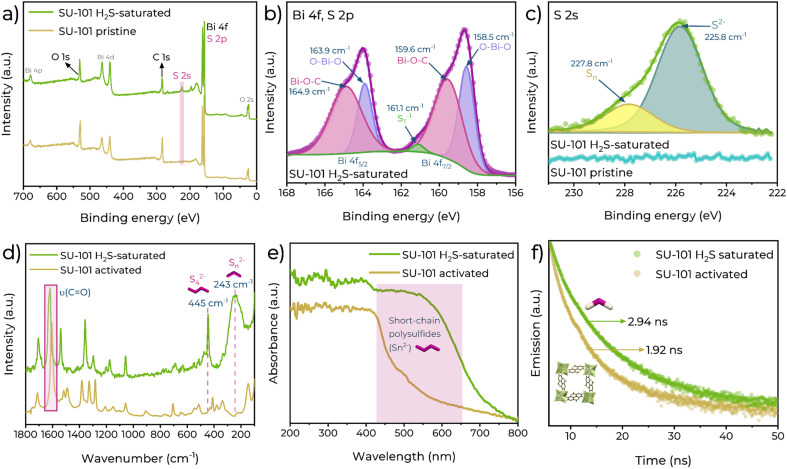
(a) XPS survey spectra of activated SU-101 (yellow) and SU-101 saturated with H_2_S (green). (b) Bi 4f/S 2p regions for XPS spectra of H_2_S-saturated SU-101. (c) Comparison of S 2s regions for XPS spectra of SU-101 saturated with H_2_S and pristine SU-101. (d) Raman spectra of activated SU-101 (yellow) and SU-101 saturated with H_2_S (green). (e) UV-vis spectra of activated SU-101 (yellow) and SU-101 saturated with H_2_S (green). (f) TRPL spectra of activated SU-101 (yellow) and SU-101 saturated with H_2_S (green).

Furthermore, the fluorescence detection of H_2_S using SU-101 was performed. First, the UV-Vis spectra of SU-101 were recorded (Fig. 3d). It was identified that at 390 nm, the material can be excited. The photoluminescence (PL) emission was measured at different excitation wavelengths (Fig. S9†). It shows a broad PL peak centred at 461 nm. The higher intensity was at the excitation wavelength of 370 nm. Subsequently, the PL emission spectra of the organic linker and SU-101 were recorded (Fig. 2a). The linker shows an emission maximum at 465 nm, whereas that of SU-101 is at 461 nm. The intense emission from the organic ligand could be related to a π* → π electronic transition, typical in conjugated bonded systems.^27^ It is observed that after the formation of SU-101, the emission from the linker is quenched, which is associated with a ligand–metal charge transfer.^28^ The LMCT process occurs when, upon excitation of the system, an electron in a π* orbital of the ligand is transferred to an empty d orbital of the metal. In the case of SU-101, Bi(iii) accepts charge from the linker (ellagic acid). After coordination, the interaction between the ligand and metal orbitals can result in a slight increase in the energy difference between the excited and fundamental states,^29^ which causes the emission maximum to slightly blueshift (toward 461 nm, shorter wavelengths).^30^ This charge transfer also decreases the intrinsic fluorescence of the ligand at the competition between two processes: the fluorescence emission from the excited state of the ligand and the non-radiative transfer of energy to the metal.^31^ As a result, the observed emission from SU-101 is not only from the ligand but is a combined phenomenon in which the charge is redistributed between the ligand and the metal.^32^

It is worth mentioning that, after the activation process, host molecules such as water, solvents or other adsorbates that may be occupying the pores of the MOF are removed. These molecules may be attenuating the fluorescence of SU-101 in its pristine state by facilitating certain non-radiative energy dissipation mechanisms, and upon removal the MOF framework is freer to emit light, resulting in higher emission efficiency. Moreover, to determine the change in the fluorescent behaviour, SU-101 was exposed to different gases (H_2_S, H_2_O, CO_2_, and air) in an in-house designed *ex situ* adsorption system (Fig. S5†). From H_2_O, CO_2_, and air exposed SU-101 samples, the PL emission spectra are similar to those of the as-synthesized SU-101 (Fig. 2c), and slight minor changes are observed after exposure to these gases. However, after the exposure to H_2_S, the PL emission spectra increased by 62.45% concerning the emission of activated SU-101 (and 289% concerning the as-synthesized emission), indicating the high selectivity for H_2_S (Fig. 2b). This PL change in intensity could be accredited to a turn-on effect due to the electronic outcome that H_2_S applies to the SU-101 framework.^33^ Then, a reproducibility test was conducted (Fig. S10†). Five independent samples of SU-101 were saturated with H_2_S, and consistently, the same turn-on performances were found.

Then, the detection properties of SU-101 under non-saturated H_2_S conditions: low H_2_S pressure. H_2_S pressure can be correlated with H_2_S detection at low H_2_S concentrations, and therefore, we needed to investigate any photoluminescence response at low H_2_S pressures (0.05 and 0.1 bar, Fig. 2b). First, an activated sample of SU-101 was exposed to 0.05 bar H_2_S, and the emission spectrum was measured. Interestingly, the broad photoluminescence peak remained centred at 461 nm, and an increase in emission intensity of approximately 13.74% with respect to the pristine sample (and 168.07% with respect to the as-synthesized emission) was observed. Later, an independent activated sample of SU-101 was exposed to 0.1 bar H_2_S, and an increase in emission intensity of approximately 37.93% with respect to the activated sample (and 225.11% concerning the as-synthesized emission) was recorded.

Furthermore, the quantitative response (sensing) of SU-101 for H_2_S was measured using a solution of H_2_S in dry THF (Fig. 2d). It is observed that an enhancement in the PL emission is a function of the increased H_2_S concentration (10–100 mM). The limit of detection (LOD) value was calculated with the following formula: LOD = 3*σ*/*m* (signal-to-noise ratio, S/N = 3), where *σ* represents the standard deviation of the initial intensity of SU-101 in dry THF and *m* is the slope of the linear fit from the experimental data (Fig. S11†). The plot of fluorescence intensity *vs.* H_2_S concentration exhibited an excellent linear correlation (*R*^2^ = 0.9628), and the LOD was calculated to be as low as 0.65 mM (∼22.16 ppm) H_2_S.

### Fluorescence mechanism

Finally, to investigate the plausible mechanism of H_2_S detection by SU-101, X-ray photoelectron spectroscopy (XPS), Raman spectroscopy, UV-vis spectroscopy, and time-resolved photoluminescence (TRPL) experiments were conducted. XPS analysis confirmed the formation of polysulphide in SU-101; a 225 eV signal appears in the survey spectra after saturation with H_2_S (Fig. 3a), which is associated with the appearance of S 2s.^34^ The Bi 4f region was inspected in detail (Fig. 3b) and shows two unequal peaks at 159.3 and 164.6 eV associated with Bi 4f_7/2_ and 4f_5/2_, displaying a separation of 5.3 eV, which is in line with the Bi^3+^ oxidation state.^35^ In addition, a small peak is observed at around 161.7 eV, and is assigned to terminal sulphur atoms (*n*-S_T_^−1^) in polysulfide compounds, indicating that a short-chain polysulfide is present after the H_2_S saturation.^36^ For the S 2s region it is observed that the spectrum of pristine SU-101 shows a flat signal (Fig. 3c), but in contrast, for the H_2_S saturated material a signal corresponding to the different sulphur states present in the polysulfides formed is observed. Two main contributions are obtained, one located at 225.8 eV and the other at 227.8 eV. The first signal at 225.8 eV has been assigned to the sulphide species (S^2−^).^37,38^ For the second peak, located at 227.8 eV, we propose that this corresponds to the polysulfide species (S_*n*_) in central positions,^39^ which could be slightly more oxidised. In addition, it has been reported that, in general terms, the lower energy signal is assigned to the more sulphur reduced form,^40^ in good agreement with our hypothesis.

Using a saturated SU-101 sample with H_2_S, a Raman spectrum (Fig. 3d) was collected. Characteristic peaks appeared at 243 cm^−1^ corresponding to different S_*n*_^2−^ species^41^ and another at 445 cm^−1^ corresponding to S_4_^2−^,^42^ confirming the chemical transformation of H_2_S to polysulfides as we previously reported,^21^ without decomposing the crystalline structure of SU-101 as confirmed by PXRD (Fig. S12†). Additionally, it is observed that the signal related to the C=O bond^43^ is shifted from 1604 to 1619 cm^−1^ after the exposure to H_2_S. This shift can be attributed to the change in the chemical environment of these groups after the interaction of H_2_S with the metal centres of Bi^3+^. Upon formation of the polysulfides, the structural rigidity of the system is modified, which alters the interactions between the metal and the linker and changes the electron density distribution in the C=O groups, resulting in the increase of the vibrational energy of the bonds, which translates into a shift towards higher frequencies.

Furthermore, changes due to the formation of polysulfides are also observed in the UV-vis spectra (Fig. 3e). The spectrum of activated SU-101 shows a weaker absorption that predominates in the range from 200 to 420 nm, while the spectrum of the H_2_S saturated material shows a more intense and extended absorption towards longer wavelengths, up to approximately 620 nm. This extension of absorbance is related to the formation of short-chain polysulfides, and it is widely reported that S_*n*_^2−^ species and polysulfide radicals have absorbances ranging from 270 to 610 nm.^39,44^ Some examples are: [S_4_]^2−^ (420 nm), [S_3_]^2−^ (340, 270 nm), [S_2_]˙^−^ (426 nm), [S_3_]˙^−^ (617 nm), and [S_4_]˙^−^ (490 nm). This corroborates that the formation of polysulfide species directly impacts the optical properties of SU-101.

Raman and XPS results indicate that, after H_2_S adsorption, polysulfides (S_*n*_^2−^ and S_4_^2−^), more rigid structure species than H_2_S, are formed within the pores of SU-101 which clearly impacts the light absorption properties and the fluorescence emission intensity of the system. Thus, the formation of polysulfides rigidifies the MOF structure, decreasing molecular motions and internal vibrations, which reduces the non-radiative energy dissipation pathways, allowing a greater proportion of the excited energy to be released in the form of fluorescence emission.^30^ Likewise, the polysulfides would be blocking the LMCT process that generated the fluorescence of SU-101.^32^ This process is interrupted by the interaction of the polysulfides formed, which can act as electron donors, with the Bi(iii) centre, and this interaction would prevent the metal centre from accepting the charge coming from the π* orbitals of the ligand. In this way, the LMCT becomes less efficient, which favours a greater amount of excited energy being released as light instead of dissipating through the LMCT, thus resulting in the turn-on effect of fluorescence after H_2_S adsorption.

Additionally, calculations of the energy gap between the HOMO–LUMO orbitals were performed using the Tauc method considering both direct and indirect transitions.^45^ For the activated material, the energy values were 2.32 eV (direct) and 2.20 eV (indirect) (Fig. S13a and S13b†), while for the material saturated with H_2_S, the values decreased to 1.83 eV (direct) and 1.73 eV (indirect) (Fig. S13c and S13d†). It is important to mention that, in molecular-type systems, the Tauc method usually underestimates or overestimates the band gap with respect to theoretical values.^46,47^ However, in this work, the Tauc method is used for comparative purposes. The reduction in the HOMO–LUMO energy suggests that the interaction between SU-101 and H_2_S causes an electronic restructuring of the material, in which the formation of new energy levels could be occurring.^48^ Actually, it has been reported that the transition energy between the HOMO and LUMO levels is related to light absorption and the smaller the HOMO–LUMO separation, the longer the absorption wavelength,^49^ which is observed in the spectrum of SU-101 exposed to H_2_S. Likewise, the decrease in the energy gap observed in the H_2_S-saturated material could be facilitating electronic transitions, which could explain the increase in absorption and the possible activation of new sensing mechanisms, such as the blocking of the LMCT mechanism.

**Fig. 4:**
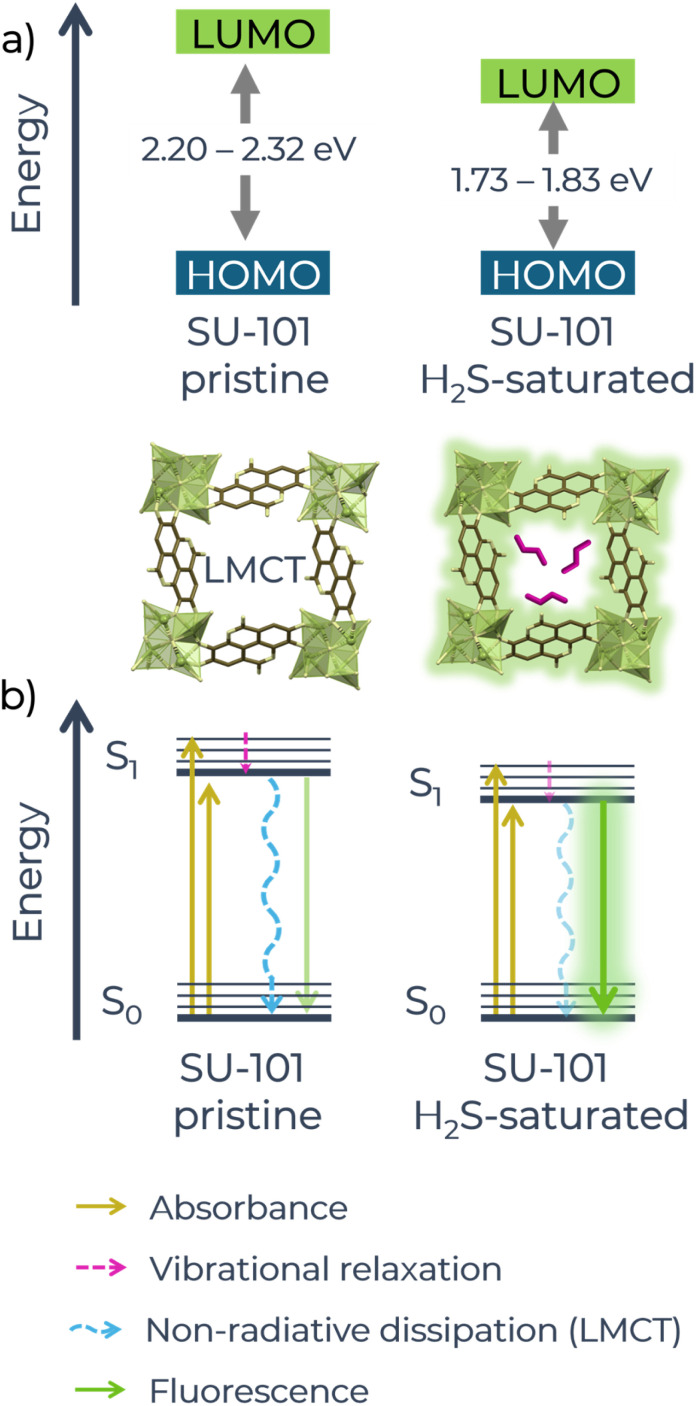
(a) Schematic of the changes in the HOMO–LUMO energy of pristine and H_2_S-saturated SU-101 material. (b) Schematic of the Jablonski diagram that would explain the changes in the fluorescence of the system.

It should be noted that the energy gap between the calculated HOMO–LUMO orbitals can only approximate the fundamental gap between the ground (S_0_) and excited (S_1_) states, as it is strongly dependent on the method used to calculate it. The separation between the S_0_ and S_1_ states is not only determined by the HOMO–LUMO gap, but is also influenced by the excitonic binding energy,^50^ which represents the interaction between the electron and the hole after excitation. However, with the data obtained from UV-vis spectroscopy, we propose a general idea of what is happening at the molecular level. By reducing the HOMO–LUMO energy (Fig. 4a), it can be deduced that the separation between the energy levels (S_0_ and S_1_) of the Jablonski diagram would also be reduced (Fig. 4b), facilitating radiative transitions from the excited state (S_1_) to the ground state (S_0_) and reducing non-radiative losses (such as vibrations). This would increase the probability of efficient radiative emission, resulting in the observed “turn-on” effect.

On the other hand, when the material is exposed to CO_2_ and H_2_O there was no significant change in the fluorescence emission, in comparison to H_2_S. We rationalise that this is because these molecules do not cause a significant chemical interaction within the pores of SU-101, in contrast to H_2_S. In other words, these molecules do not interact with the material in a way that modifies its structure or affects the LMCT process that determines the fluorescence emission in the material. As a result, the fluorescence of the MOF remains virtually unchanged when exposed to these gases, without producing the “turn-on” or increase in fluorescence that is observed with H_2_S.

Finally, TRPL experiments were carried out using a 370 nm picosecond-pulsed LED as the excitation source and performed on an activated sample of SU-101 and an H_2_S-saturated sample. The PL decay spectra was measured at 461 nm (Fig. 3f), showing that the average decay lifetimes increased upon H_2_S exposure (Table S3†). The average fluorescence lifetime of the activated sample was 1.92 ns, while the lifetime of the H_2_S saturated sample was 2.94 ns. To provide an explanation for this increase in fluorescence time, the components (*τ*) and their contributions to the total fluorescence time can be examined.^51–53^

In both cases, *τ*_1_ is the fastest component (*τ*_1(act)_ = 0.09 ns and *τ*_1(H2S)_ = 0.06 ns), since it has an extremely short lifetime, which suggests that it represents a fast relaxation process, probably non-radiative;^54^ for the activated sample its relative contribution is *a*_1(act)_ = 18.96%, while for the saturated sample its contribution decreases to *a*_1(H2S)_ = 5.48%, which would be manifesting that these fast non-radiative relaxation processes decreased considerably after saturation with H_2_S. On the other hand, *τ*_2_ is a component with an intermediate lifetime (*τ*_2(act)_ = 0.87 ns and *τ*_2(H2S)_ = 1.18 ns), which could represent a mixture of radiative and non-radiative processes; its contribution has a slight increase after saturating the sample (from *a*_2(act)_ = 34.15% to *a*_2(H2S)_ = 37.44%) which indicates that the intermediate decay processes have gained in efficiency, which may be due to the stiffening of the MOF framework after H_2_S adsorption, which reduces the non-radiative losses. Finally, the *τ*_3_ and *τ*_4_ components with slow times represent different radiative mechanisms, *i.e.*, more efficient processes in light emission.^38,39^ In both cases their contributions increase after saturation with H_2_S, for *τ*_3_ from *a*_3(act)_ = 36.78% to *a*_3(H2S)_ = 41.38% and for *τ*_4_ from *a*_4(act)_ = 10.11% to *a*_4(H2S)_ = 15.70%, the increase in the value of these two components is an indication that the structural stiffening induced by the formation of polysulfides and the LMCT process block generates the reduction of internal vibrations and non-radiative mechanisms, as represented in the Jablonski diagram in Fig. 4b, introducing a higher degree of stability in the system, allowing a longer and more efficient radiative emission.

### Polysulfides interaction on SU-101: electronic structure calculations

In order to examine the interaction of polysulfide species formed inside the SU-101 structure from an atomistic perspective, the spatial structure of the MOF is obtained from the CIF file previously reported by Grape *et al.*^21^ To start this geometry, we build a supercell of size 2 × 2 from the unit cell. The representative optimised geometry of the SU-101 is shown in Fig. S13(a).† The relaxed structure corresponds to a supercell with dimensions of *a* = *b* = 18.62 Å and *c* = 11.09 Å. This configuration was designed to create the characteristic micropore of SU-101, capable of stabilising small polysulfide species of varying sizes. The crystal structure is depicted in Fig. S13(b),† where the formation of octahedra around the Bi(iii) metallic centres is evident. Additionally, the partial density of states (PDOS), shown in Fig. S13(c),† shows that the electronic contributions from C and O atoms are concentrated near the Fermi level, while Bi(iii) atoms exhibit minimal contributions just above it. These findings suggest that charge transfer processes are primarily influenced by the ligand atoms, with the metal centres playing a comparatively minor role.

**Fig. 5:**
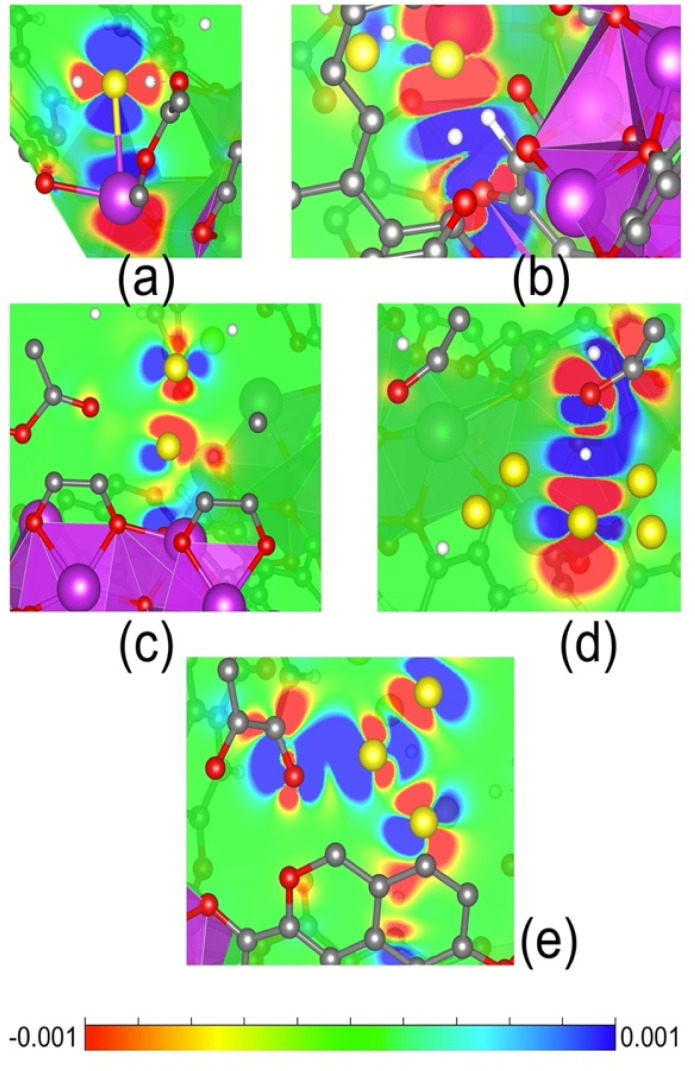
2D plot of the electron density difference of (a) H_2_S/SU-101, (b) H_2_S_2_/SU-101, (c) H_2_S_4_/SU-101, (d) H_2_S_6_/SU-101, and (e) H_2_S_8_/SU-101. Blue regions of 2D slices represent sites from which the electronic charge was depleted, and the red areas refer to the locations where the charge is accumulated (an isovalue of 0.001 e Å^−3^).

In order to investigate the stability and potential formation of polysulfide species, we examined small clusters of H_2_S_*x*_ (*x* = 1, 2, 4, 6, 8) of different sizes. The optimised molecular structures are shown in Fig. S14,† where the average bond length between different clusters is approximately 2.09 Å (S–S). Geometry relaxed calculations were carried out for all H_2_S_*x*_ clusters inside the pore of SU-101. The final configurations are illustrated in Fig. S15.† For the H_2_S molecule that interacts with the surface of SU-101, it demonstrates an interaction with the metallic centre of Bi(iii) at a distance of 3.12 Å, while simultaneously sharing its proton with a nearby oxygen atom (see Fig. S15(a)†). When analysing the interactions with polysulfide species, the configurations after geometry relaxation are shown in Fig. S15(b–e).† It can be observed that the H_2_S_*x*=2–8_/SU-101 molecules do not interact directly with the metallic centres of Bi(iii). In these cases, the polysulfide molecules lose their initial configuration but remain confined within the pore of SU-101. In particular, the closest S–O interaction is observed in the H_2_S_8_/SU-101 configuration, with a distance of 2.34 Å, while the H_2_S_4_/SU-101 configuration exhibits the largest S–O distance of 3.56 Å. This behaviour is attributed to variations in the attraction between the molecule and the micropore surface. For H_2_S_2_/SU-101 and H_2_S_6_/SU-101, the S–O interaction distances are relatively similar, measuring 3.00 Å and 3.06 Å, respectively. These findings reveal a correlation between the size of certain polysulfides and their interaction strength with the surface of SU-101. To further understand these interactions, we performed adsorption energy calculations (*E*_ads_), providing insight into the energetic stability of the studied configurations. *E*_ads_ is obtained from the following expression: *E*_ads_ = *E*_int_ − (*E*_SU-101_ + *E*_H2S*x*_), where *E*_int_ corresponds to the total energy of the system adsorbate/SU-101 surface; ESU-101 is the total energy of the relaxed structured MOF, and *E*_H2S*x*_ is the total energy of the polysulfide species under study. The negative values are related to the exergonic interaction between the adsorbate and the surface of SU-101. The results of the *E*_ads_ values are reported in Table S4.† All *E*_ads_ values are exergonic, indicating a favourable interaction of the adsorbates within the pore of SU-101. Particularity, the H_2_S/SU-101 configuration shows *E*_ads_ = −1.30 eV, which corroborates the formation of H_2_S-saturated SU-101. It is worth mentioning that from H_2_S_4_ (−0.87 eV), H_2_S_6_ (−1.01 eV), and H_2_S_8_ (−1.78 eV) molecules the *E*_ads_ values begin to increase; this behaviour corroborates the presence of polysulfide molecules of different sizes within the micropore of SU-101. To understand the charge transfer mechanism behind the interaction between the adsorbates and the SU-101 material, the density difference isosurfaces (Δ*ρ*) were plotting according to: Δ*ρ* = *ρ*_total_ – *ρ*_adsorbate_ − *ρ*_surface_, where *ρ*_total_ is the electron density of the system adsorbate/SU-101 surface, *ρ*_adsorbate_ is the electron density of the polysulfide species under study, and *ρ*_surface_ refers to the electron density of the SU-101 structure. Fig. 5 represents the Δ*ρ* for each interaction under study. In the case of the H_2_S/SU-101 configuration, the regions of electron density removal (Fig. 5a) between the S–Bi atoms are evident, this mechanism favours a high *E*_ads_ value. The same mechanism is observed for the case of the H_2_S_8_/SU-101 interaction between the S–O atoms (Fig. 5d). The systems H_2_S_2_/SU-101 and H_2_S_6_/SU-101 show charge accumulation (red colour) on the S atoms and charge density removal between the O–H atoms (blue colour). The H_2_S_4_/SU-101 interaction presents a characteristic behaviour because charge accumulations are present between the S atom and the ligand fragment. The accumulation of charge in S_2–8_ small polysulfides is evident, which can be correlated with the nature of the charge species. Thus, Δ*ρ* shows evidence of how the organic fragments of the MOF material are involved in the interaction process. Finally, TD-DFT calculations were performed to simulate UV-vis absorption spectra, as shown in Fig. S16.† The results reveal that the behaviour and shape of the spectra are largely influenced by the SU-101 material. In particular, the maximum absorption peak is observed when H_2_S_8_ interacts with SU-101. This indicates that the size of the polysulfides within the SU-101 structure significantly affects its optical response.

## Conclusions

In summary, the detection properties of a structurally stable Bi(iii)-based MOF material, SU-101, were investigated by fluorescence experiments. These demonstrated a significant change in the emission spectra after H_2_S adsorption (chemical transformation to polysulfides), with a clear H_2_S detection selectivity (over H_2_O and CO_2_) and a reproducible H_2_S response when SU-101 was exposed to 0.05 bar H_2_S, 0.1 bar H_2_S and when this material was saturated with H_2_S (*i.e.*, 1 bar H_2_S). Remarkably, the limit of H_2_S detection (LOD) was calculated to be as low as 0.65 mM (∼22.16 ppm). The fluorescence enhancement is attributed to the chemical transformation of H_2_S into polysulfides within the pores of the material confirmed by Raman and XPS experiments, which induced a rigidification in the structure of the system, decreasing the molecular vibrations. In addition, the electron-donor character of the polysulfides formed generates interactions with the Bi(iii) metal centre, creating a blockage in the LMCT process, which decreased certain non-radiative processes, affording a “turn-on” effect in the fluorescent emission. Analysis of the UV-vis spectra of SU-101 before and after H_2_S adsorption reveals significant changes in the optical properties of the material. The reduction of the bandgap, determined by the Tauc method, shows that the polysulfide formation introduces new discrete energy levels within the original gap of the material, allowing electronic transitions at lower energies, which facilitates light absorption in a broader region of the spectrum. Time-resolved photoluminescence (TRPL) experiments provided further insight into the detection mechanism, confirming that the increased fluorescence lifetime observed after H_2_S exposure is due to the reduction of non-radiative dissipation mechanisms. DFT calculations shows that the adsorption energy increases with the size of the polysulfide chain, as observed for the interaction of H_2_S_4_, H_2_S_6_, and H_2_S_8_ molecules within the SU-101 material. Overall, this study postulates this chemically stable Bi(iii)-based MOF material, SU-101, as a promising candidate for H_2_S detection.

## Data availability

All data is available in the main text and in the ESI.†

## Conflicts of interest

There are no conflicts to declare.

## Author contributions

Valeria B. López-Cervantes: methodology, formal analysis, investigation, data curation, writing – original draft. Juan L. Obeso: methodology, formal analysis, investigation, writing – original draft. J. Gabriel Flores: methodology, formal analysis, investigation, writing – original draft. Aída Gutiérrez-Alejandre: validation, supervision, resources. Raul A. Marquez: investigation, validation, formal analysis. José Antonio de los Reyes: resources, visualization. Catalina V. Flores: investigation. N. S. Portillo-Vélez: investigation. Pablo Marín-Rosas: investigation. Christian A. Celaya: computational calculations. Eduardo González-Zamora: resources, visualization. Diego Solis-Ibarra: conceptualization, resources, project administration, supervision, visualization. Ricardo A. Peralta: conceptualization, resources, supervision, writing – review & editing, visualization. Ilich A. Ibarra: conceptualization, resources, funding acquisition, project administration, supervision, writing – review & editing, visualization.

## Supplementary Material

SC-OLF-D4SC07144A-s001
