# Supplementary material for: Formation of polysulfides as a smart strategy to selectively detect H2S in a Bi(iii)-based MOF material
Source: Chem Sci. 2025 Feb 19;16(13):5483–92. doi: 10.1039/d4sc07144a (PMC11853078; doi:10.1039/d4sc07144a)
Supplement: SC-016-D4SC07144A-s001 [file SC-016-D4SC07144A-s001.pdf]

Supplementary Information (SI) for Chemical Science.
This journal is © The Royal Society of Chemistry 2025

Supporting Information for:

Formation of polysulfides as a smart strategy to selectively detect H_2S in a Bi(III)-based MOF material

Valeria B. López-Cervantes,[a†] Juan L. Obeso,[a,b†] J. Gabriel Flores,[c,d†] Aída Gutiérrez-Alejandre,[e] Raul A. Marquez,[f] José Antonio de los Reyes,[d] Catalina V. Flores,[a,b] N. S. Portillo-Vélez,[g] Pablo Marín-Rosas,[g] Christian A. Celaya,[h] Eduardo González-Zamora,[g] Diego Solis-Ibarra,*[a] Ricardo A. Peralta*[g] and Ilich A. Ibarra*[a,i]

[a.] *Laboratorio de Fisicoquímica y Reactividad de Superficies (LaFReS), Instituto de Investigaciones en Materiales, Universidad Nacional Autónoma de México, Circuito Exterior s/n, CU, Coyoacán, 04510, Ciudad de México, México. Ilich A. Ibarra: Email: argel@unam.mx*

[b.] *Instituto Politécnico Nacional, CICATA U. Legaria, Laboratorio Nacional de Ciencia, Tecnología y Gestión Integrada del Agua (LNAgua), Legaria 694, Irrigación, 11500, Miguel Hidalgo, CDMX, México.*

[c.] *Área de Química Aplicada, Departamento de Ciencias Básicas, Universidad Autónoma Metropolitana-Azcapotzalco, 02200, Ciudad de México, México*

[d.] *Departamento de Ingeniería de Procesos e Hidráulica, División de Ciencias Básicas e Ingeniería, Universidad Autónoma Metropolitana-Iztapalapa, 09340, Ciudad de México, México.*

[e.] *UNICAT, Departamento de Ingeniería Química, Facultad de Química, Universidad Nacional Autónoma de México, 04510 Ciudad de México, México.*

[f.] *Department of Chemistry, The University of Texas at Austin, Austin, Texas 78712, United States.*

[g.] *Departamento de Química, División de Ciencias Básicas e Ingeniería. Universidad Autónoma Metropolitana (UAM-I), 09340, México. Ricardo A. Peralta: Email: rperalta@izt.uam.mx.*

[h.] *Centro de Nanociencias y Nanotecnología, Universidad Nacional Autónoma de México, Km 107 Carretera Tijuana-Ensenada, Ensenada, B.C., C.P. 22800, Mexico.*

[i.] *On Sabbatical as "Catedra Dr. Douglas Hugh Everett" at Departamento de Química, Universidad Autónoma Metropolitana-Iztapalapa, Avenida San Rafael Atlixco 186, Leyes de Reforma 1ra Sección, Iztapalapa, Ciudad de México 09310, México.*

Table of contents

S1. Experimental details ……………………………..……………………..…………………..… S3

S2. Results and Discussion ……….………………………….………………………………. S4

S3. References ……………………………..……………………………..………………...…. S16

S1. Experimental details

Materials

Bismuth (III) acetate ($(CH_3CO_2)_3Bi$, 99.99 %), Ellagic acid (HPLC ≥ 95 %), Acetic acid glacial (CH_3CO_2H, 99 %) were supplied by Sigma-Aldrich. All reagents, gases, and solvents were used as received from commercial suppliers without further purification.

Analytical instruments

Powder X-Ray Diffraction Patterns (PXRD)

PXRD was recorded on a Rigaku Diffractometer, Ultima IV, with Cu-Kα1 radiation ($\lambda = 1.5406$ Å) using a nickel filter. The patterns were recorded in the range 2–50° 2θ with a step scan of 0.02° and a scan rate of 0.05° min^{-1}.

Fourier-transform infrared spectroscopy (FT-IR)

FT-IR spectra were obtained in the range of 4000-500 cm^{-1} on a Shimadzu IRTracer-100 spectrometer with a Golden Gate Single Reflection diamond ATR cell.

Thermal gravimetric analysis (TGA)

TGA was performed using a TA Instruments Q500HR analyzer under an N_2 atmosphere using the high-resolution mode (dynamic rate TGA) at a scan rate of 5 °C min^{-1}, from room temperature to 800 °C.

Solid-state ultraviolet-visible spectroscopy (UV-Vis)

Absorption measurements were performed from 200-800 nm using a Shimadzu spectrophotometer UV-2600 equipped with an ISR-2600Plus integrating sphere and a $BaSO_4$ blank.

X-ray Photoelectron Spectroscopy (XPS)

It was conducted with a PHI VersaProbe 4 instrument using a nonmonochromatic Al Kα source (1486.6 eV) and the charge neutralizer. The instrument's base pressure was ~10^{-9} torr. High-resolution spectra were collected over an analysis area of ~250 × 250 μm^2 using a pass energy of 10 eV. Binding energy was calibrated using the C 1s peak for adventitious hydrocarbons at 284.8 eV. Data analysis was performed using CasaXPS software.

S2. Results and Discussion

Characterization of SU-101

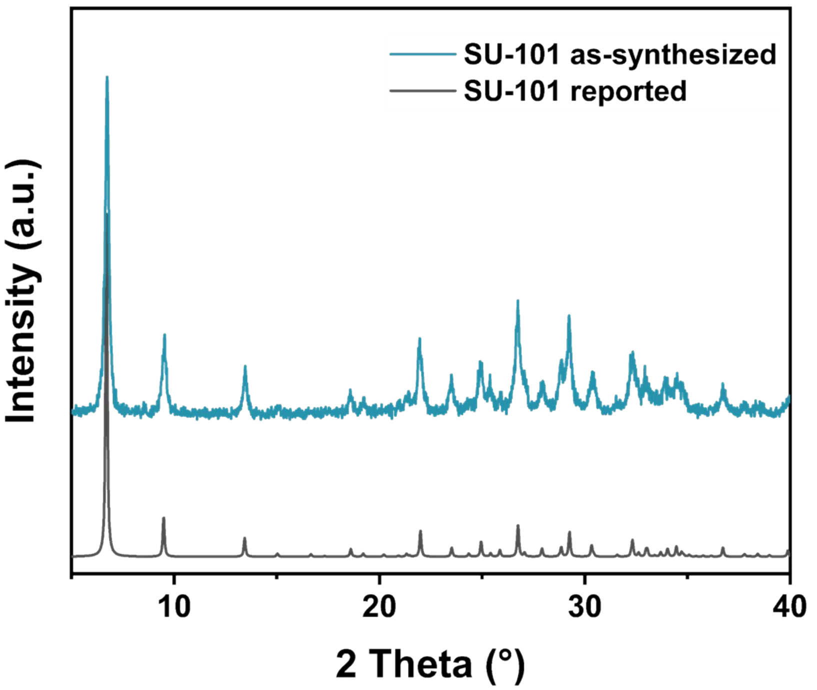

Figure S1. PXRD pattern of SU-101 reported and SU-101 as-synthetize

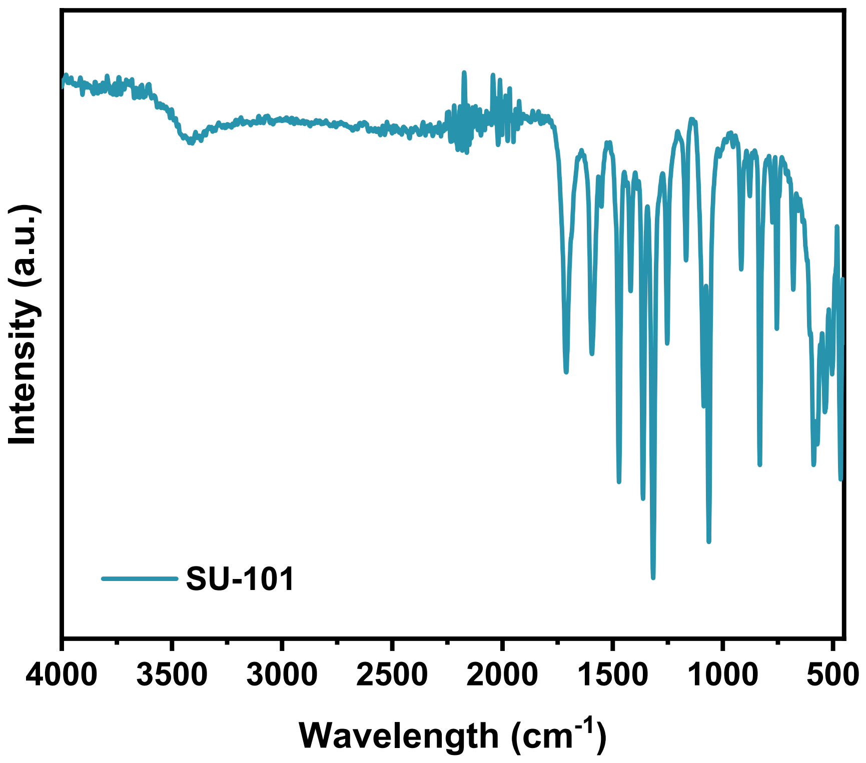

Figure S2. FTIR spectra of SU-101 as-synthetized.

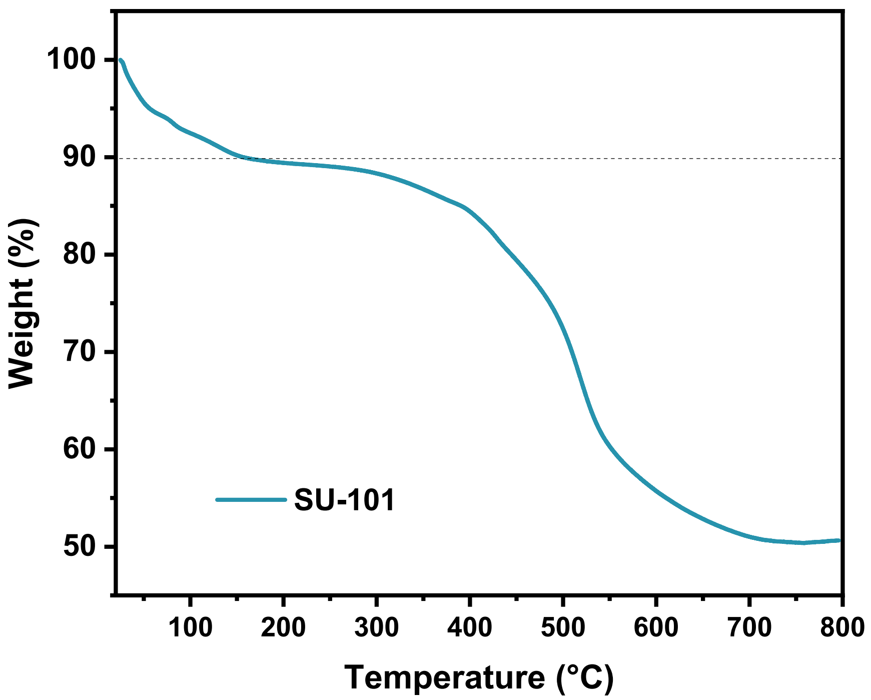

Figure S3. TGA profile of SU-101 as-synthetized.

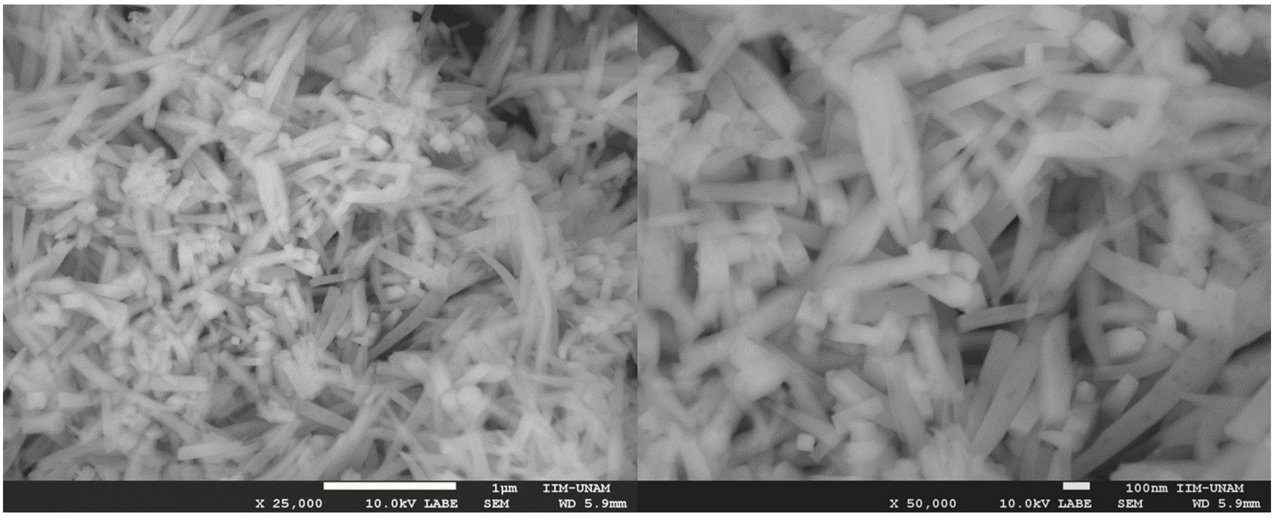

Figure S4. SEM images of SU-101 as-synthetized.

H_2S saturation experiments

The system (Figure S5) contains two principal parts:

A. The gas generator, in which Fe_2S_3 is added to a two-neck ball flask [1], one of which is capped with a rubber stopper through which concentrated HCl is injected with a glass syringe [2], while the other port is connected to the saturation chamber.
B. The saturation chamber, made of a round flask [3], is connected to a vacuum line [4] and a vacuum line [4]. vacuum line [4] and a pressure gauge [5].

To start the process, a sample of about 15 mg in a 1.5 mL glass vial was activated in a sand bath with N_2 flow at 120 °C under vacuum for 12 h. The vial was then placed in the saturation chamber, and the system was evacuated with a vacuum line. Next, H_2S gas was generated by dripping concentrated HCl over Fe_2S_3, the sample was left continuously exposed to the gas for 3 hours.

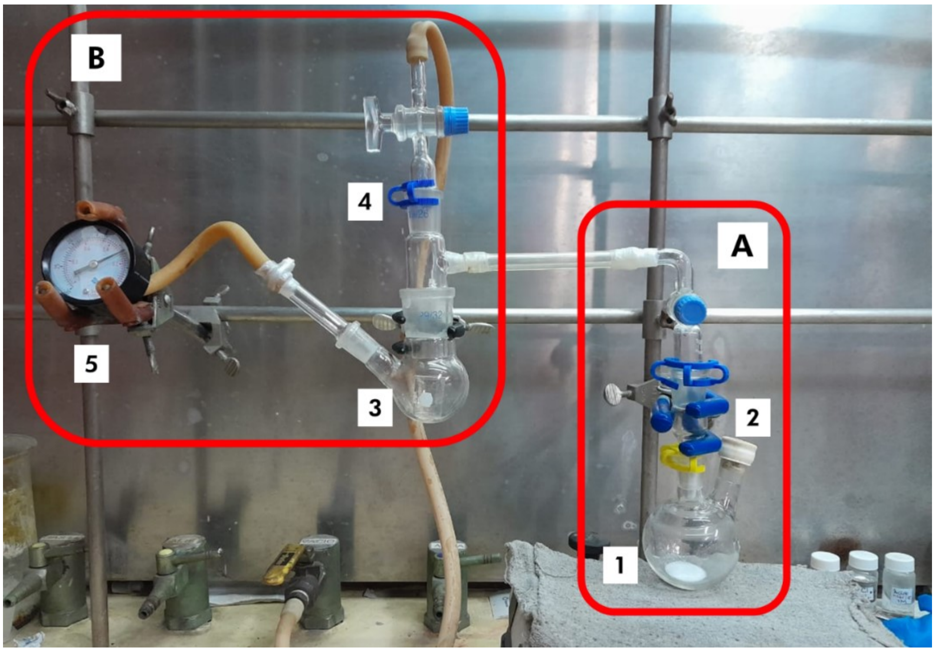

Figure S5. *in-situ* H_2S homemade system.

H_2S breakthrough experiments

H_2S experiments were made using a HP 5890 GC, by continuous injections of the system exhaust, of each injection we obtained a chromatogram. From the corresponding chromatogram

we integrate the H_2S signal to obtain its quantity. Knowing the H_2S concentration from the feed, we can calculate the H_2S concentration in each one of the injections, as the saturation concentration is the original feed concentration. Dynamic breakthrough experiments were carried out in a home-made system.

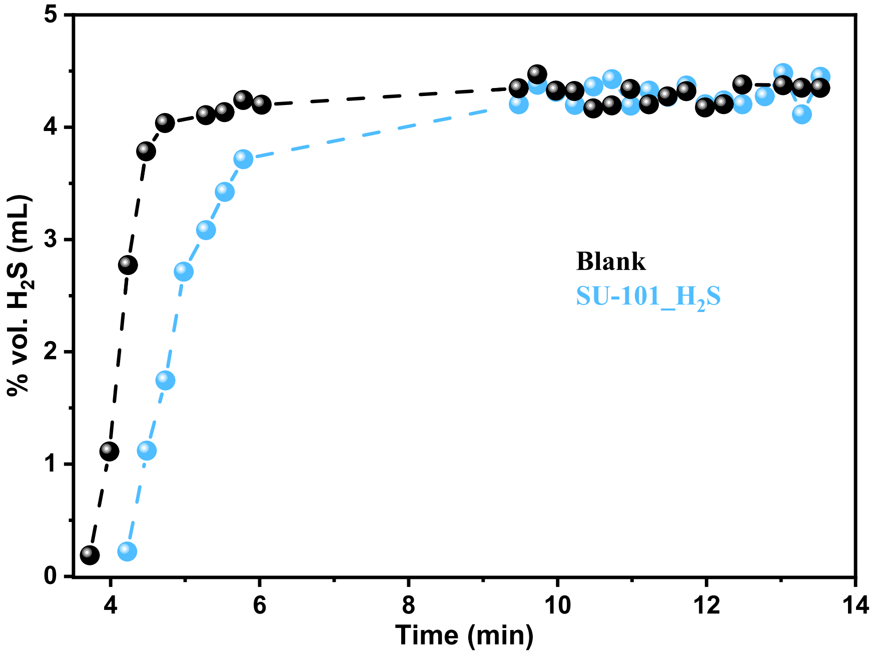

Figure S6. Breakthrough curve of H_2S adsorption by SU-101 at 25 °C and 1 bar.

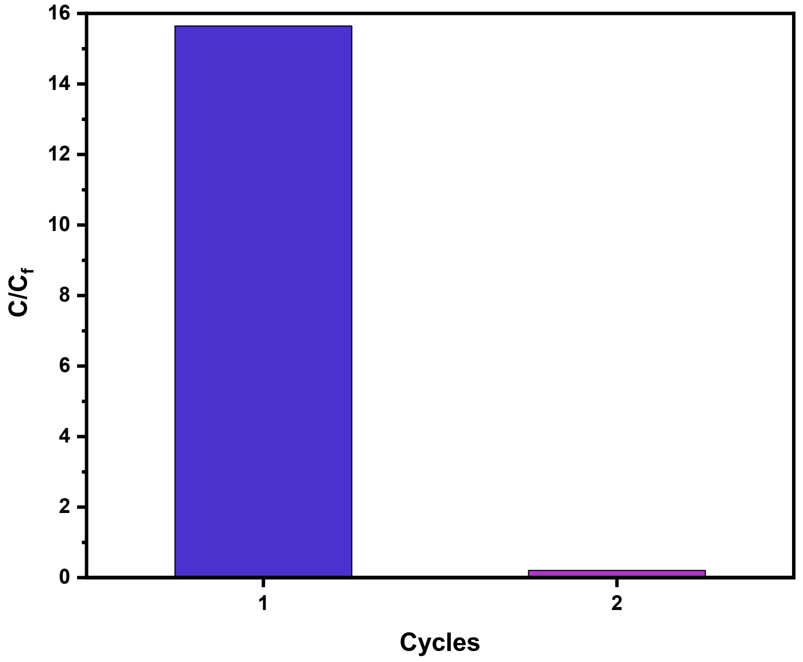

Figure S7. Comparative H_2S adsorption capacities for two cycles.

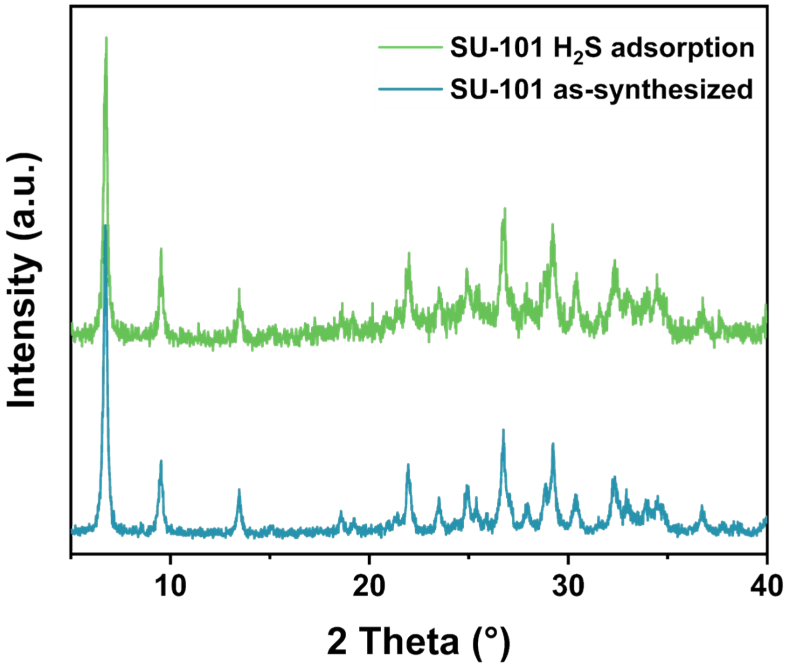

Figure S8. PXRD patterns of SU-101 as-synthetized and SU-101 after H_2S adsorption.

Table S1. Evolution of SU-101 as a function of contact time with H_2S, quantifiable in GC.

Time (s)	H_2S uptake (mmol g^{-1})	Image
120	0.00	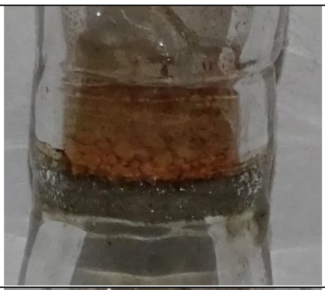
145	0.06	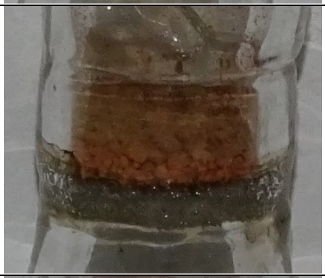
170	0.15	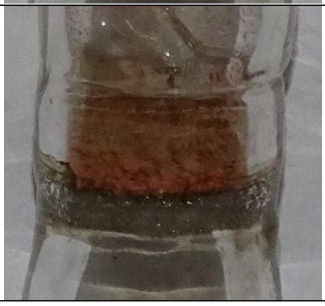

195	0.24	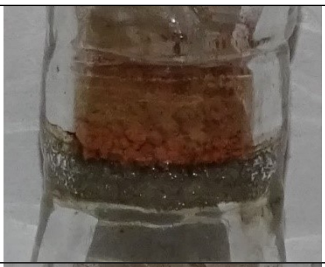
220	0.33	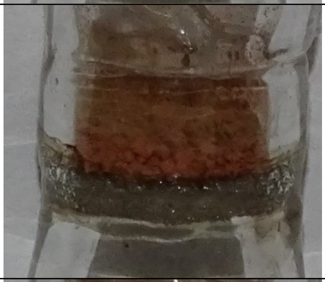
245	0.44	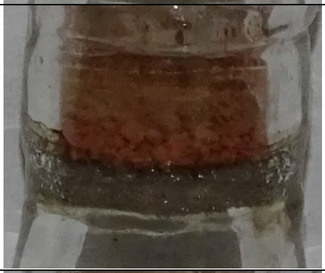
270	0.46	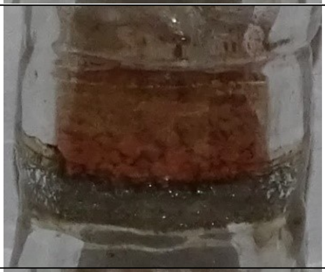
670	0.73	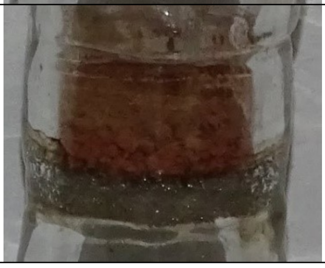

Fluorescence experiments

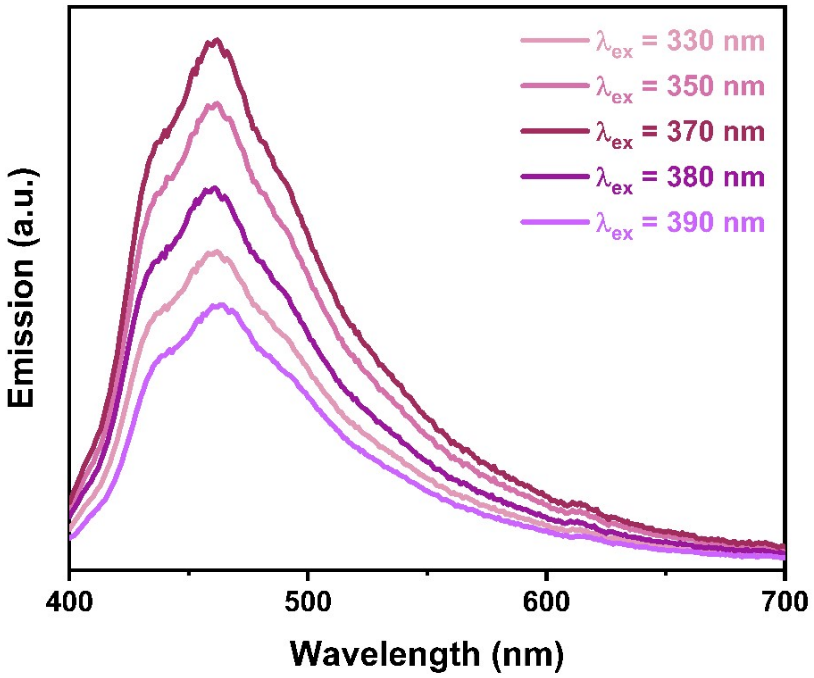

Figure S9. Solid-state emission spectra of activated SU-101 at different excitation wavelengths.

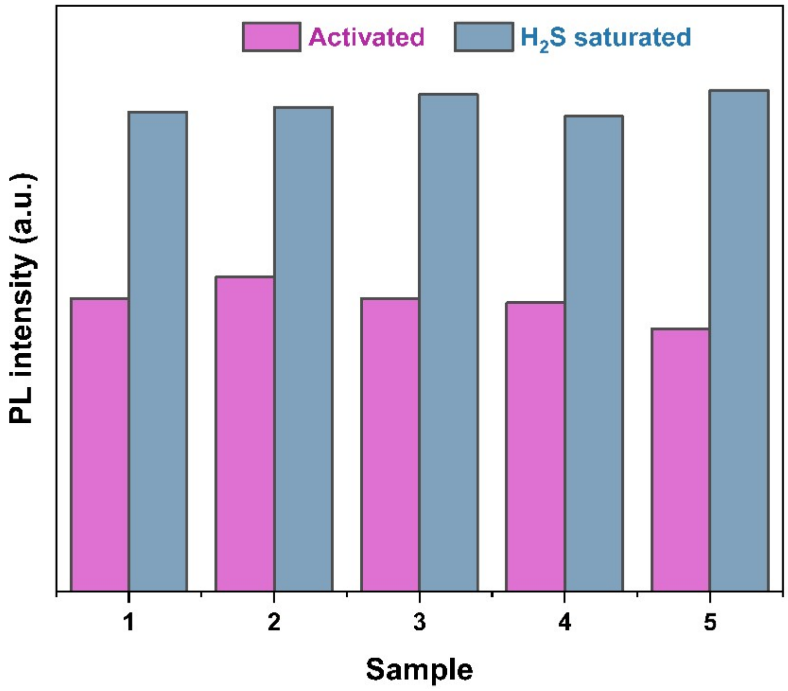

Figure S10. Fluorescence emission of five independent samples activated and saturated with H_2S.

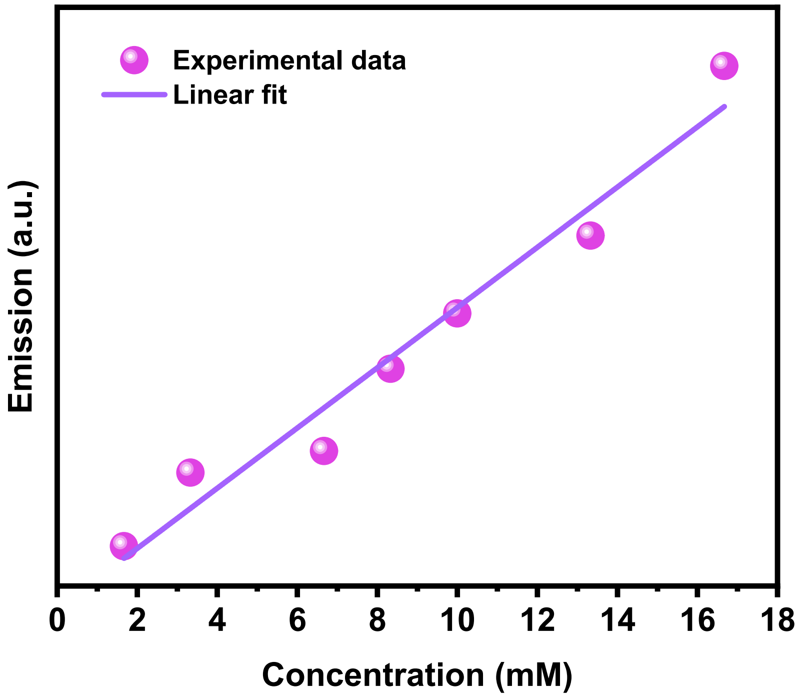

Figure S11. Determination of LOD.

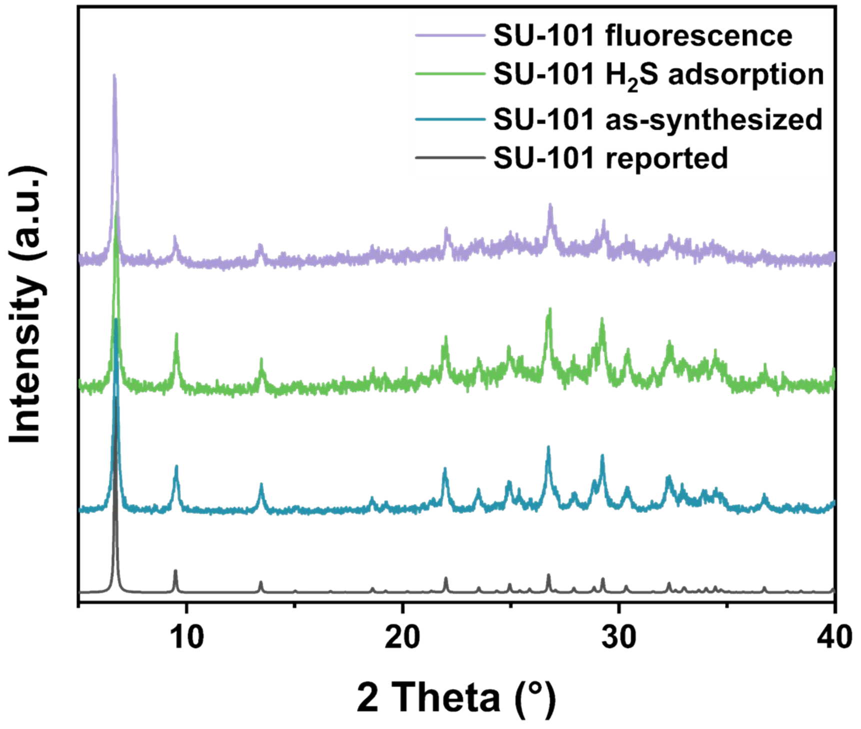

Figure S12. PXRD patterns of SU-101 reported, as-synthetized, after H_2S in our home-made in situ device, and after the florescence test.

Tauc plots for the determination of the energies between HOMO-LUMO orbitals by direct and indirect method

The determination of the energy between the HOMO-LUMO orbitals of the activated and H_2S-saturated SU-101 material, were performed by constructing Tauc plots using solid-state UV-visible spectroscopy data.[S2] Tauc plots in Figure S13, allow the assessment of the type of electronic transition present, either a direct or indirect transition, based on the analysis of the optical absorption of the material.

The following relationships were used for this assessment:

- Direct transitions: $(\alpha h\upsilon)^2 \propto (h\upsilon - E_{gap})$
- Indirect transitions: $(\alpha h\upsilon)^{\frac{1}{2}} \propto (h\upsilon - E_{gap})$

Where α is the absorption coefficient, $h\upsilon$ is the photon energy, and E_{gap} represents the HOMO-LUMO energy gap. By extrapolating the linear region of the Tauc plot to α=0, the E_{gap} value for each transition type is obtained.

The values obtained for the direct and indirect transitions are shown in Table S2.

Table S2. HOMO-LUMO energy values considering direct and indirect transitions calculated from the Tauc method for the activated, and H_2S-saturated SU-101 samples.

Sample	Direct (eV)	Indirect (eV)
**SU-101 activated**	2.32	2.20
**H_2S saturated**	1.83	1.73

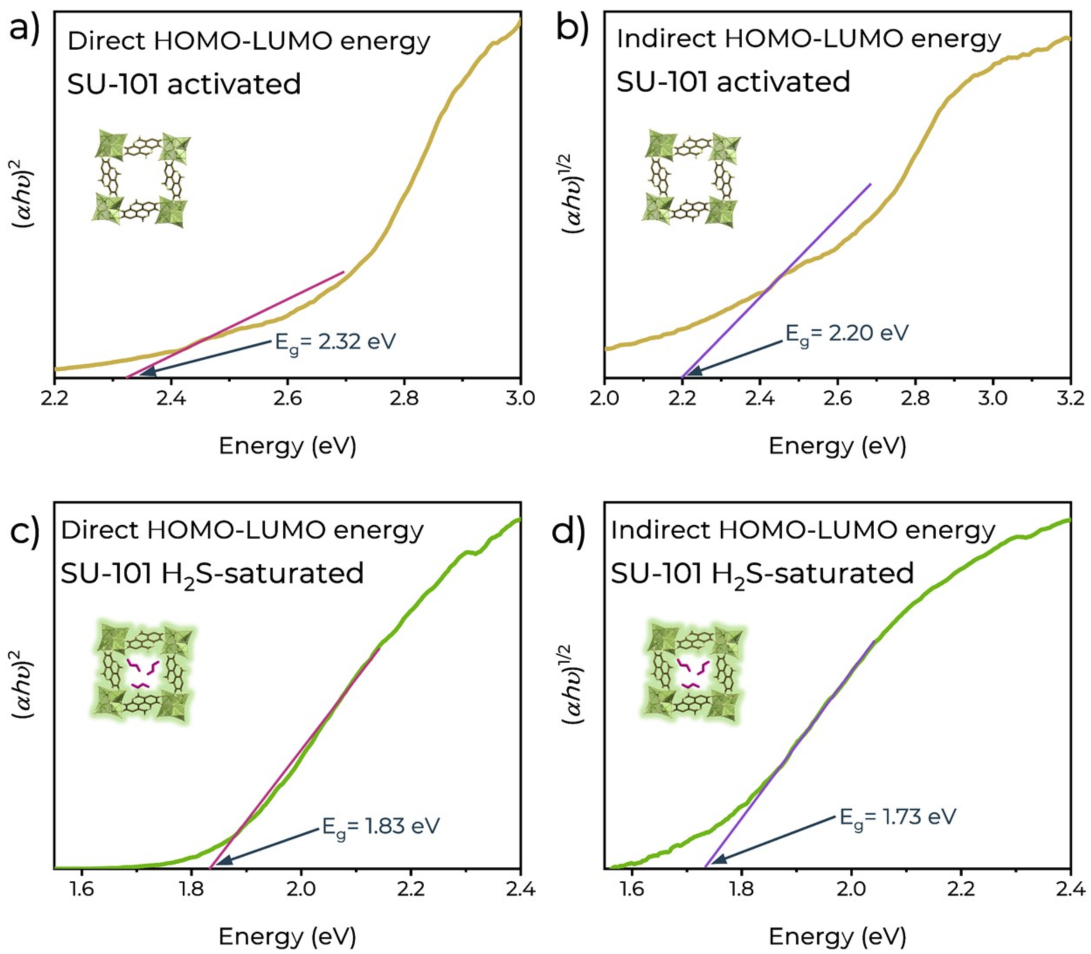

Figure S13. Tauc plots considering direct and indirect transitions for (a) and (b) activated SU-101 (yellow), and (c) and (d) saturated with H_2S (green).

TRPL experiments

Fluorescence lifetimes were determined from the TPRL spectra. The data obtained from the decay spectra were fitted in Fluoracle software, using a multi-exponential equation (Equation 1) to describe the fluorescence emission decay curve:[S3]

$$R(t) = B_1 e^{\left(\frac{-t}{\tau_1}\right)} + B_2 e^{\left(\frac{-t}{\tau_2}\right)} + B_3 e^{\left(\frac{t}{\tau_3}\right)} + B_4 e^{\left(\frac{-t}{\tau_4}\right)}$$

where R(t) represents the fluorescence intensity as a function of time, B_1, B_2, B_3 and B_4 are the amplitudes of the respective decay components, and τ_1, τ_2, τ_3 and τ_4 are the lifetimes of the different components.

Table S3. Lifetimes of the activated and saturated samples.

SU-101	τ_1 (ns)	a_1	τ_2 (ns)	a_2	τ_3 (ns)	a_3	τ_4 (ns)	a_4	Lifetime (ns)
**Activated**	0.0866	0.1896	0.8685	0.3415	2.1830	0.3678	7.9120	0.1011	**1.9158**
**H_2S saturated**	0.0597	0.0548	1.1787	0.3744	2.8721	0.4138	8.2971	0.1570	**2.9357**

Electronic structure calculations

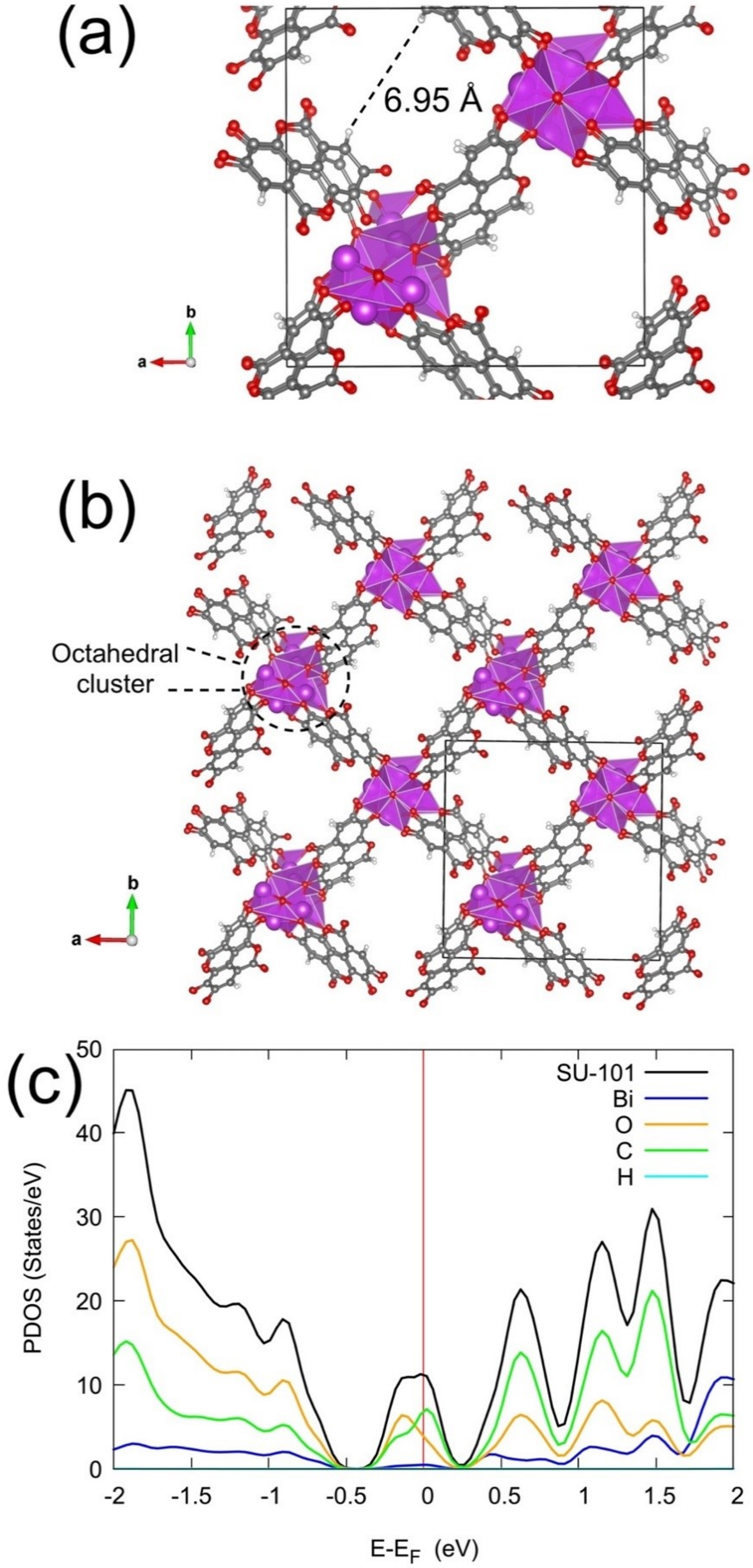

Figure S13. (a). The supercell 2 x 2 relaxed, (b) periodic conditions perspective of SU-101 crystal structure, and (c) partial density of states (PDOS) of SU-101 structure.

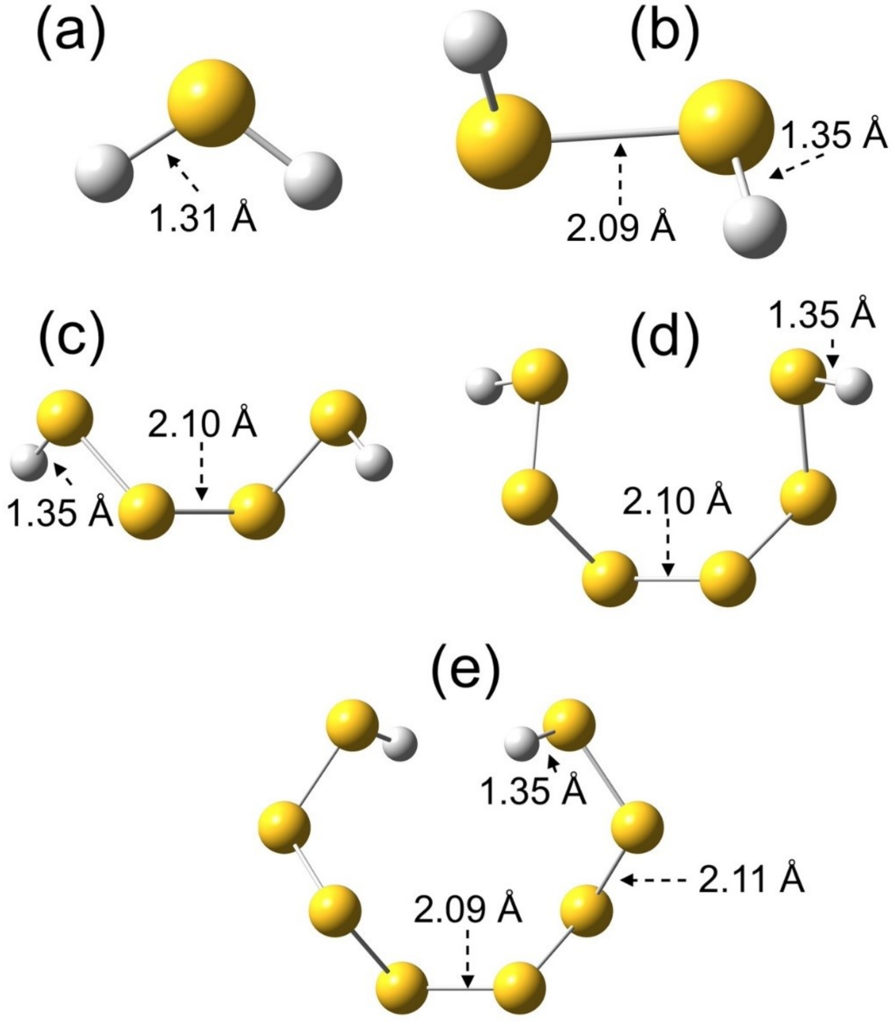

Figure S14. Optimized geometries corresponding of (a) H_2S molecule and polysulfide species to (b) H_2S_2, (c) H_2S_4 (d)H_2S_6, (e) H_2S_8.

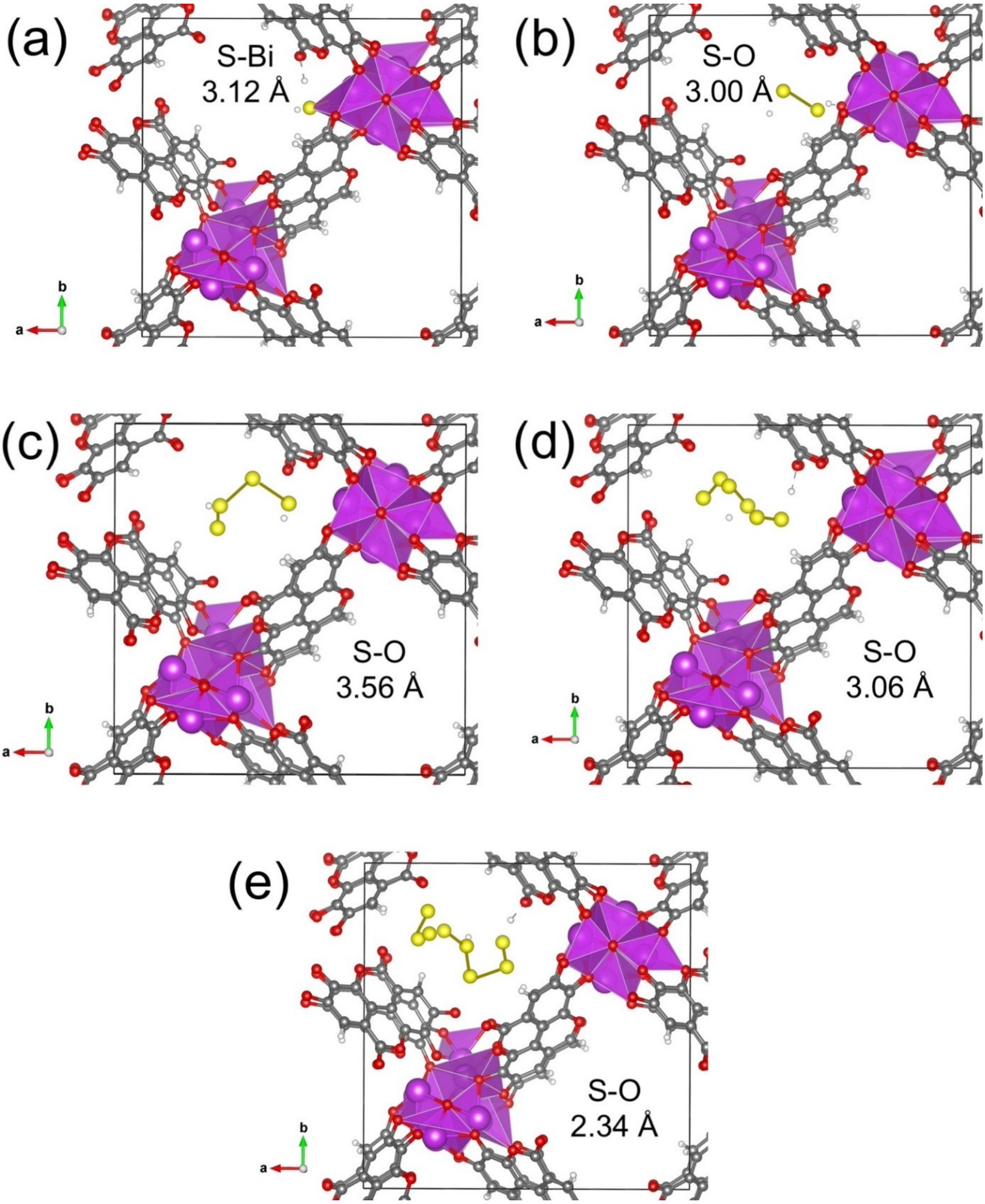

Figure S15. Optimized geometries corresponding H_2S molecule and polysulfide interaction into SU-101 structure: (a) H_2S/SU-101, (b) H_2S_2/SU-101, (c) H_2S_4/SU-101, (d)H_2S_6/SU-101, (e) H_2S_8/SU-101.

Table S4. Adsorption energies (E_{ads}) given in the interaction of the SU-101 structure interacting with H_2S molecule and polysulfide under study.

System	E_{ads} (eV)
H_2S/SU-101	-1.30
H_2S_2/SU-101	-1.50
H_2S_4/SU-101	-0.87
H_2S_6/SU-101	-1.01
H_2S_8/SU-101	-1.78

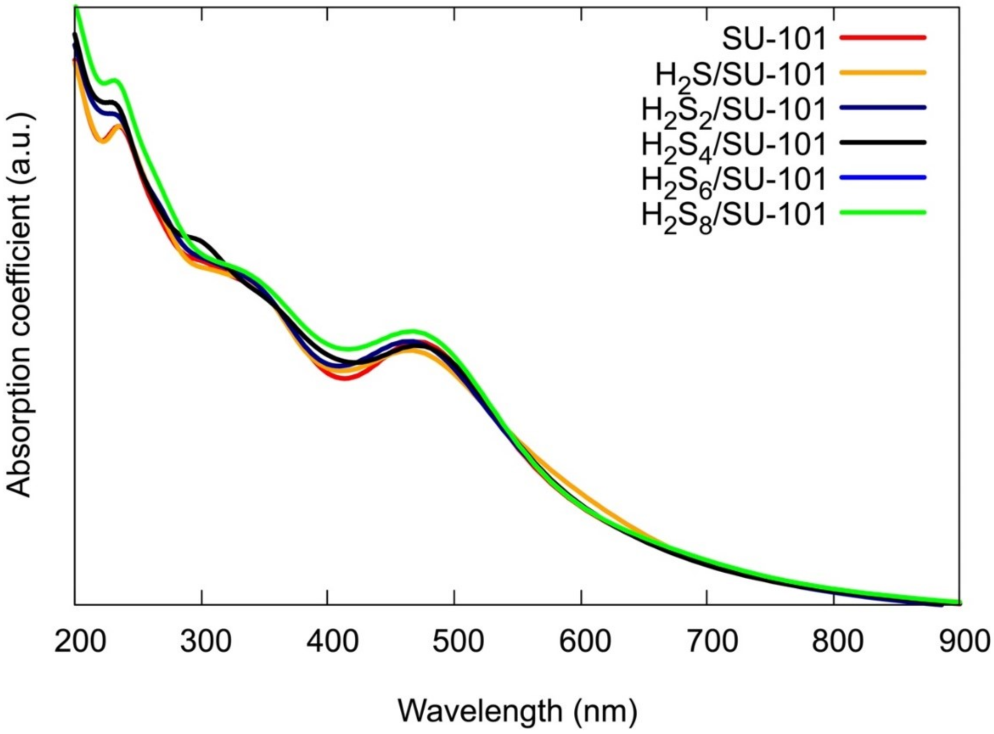

Figure S16. Simulated absorption spectra of the SU-101 structure interacting with H_2S molecule and polysulfide under study.

S3. References

(1) Grape, E. S.; Flores, J. G.; Hidalgo, T.; Martínez-Ahumada, E.; Gutiérrez-Alejandre, A.; Hautier, A.; Williams, D. R.; O'Keeffe, M.; Öhrström, L.; Willhammar, T.; Horcajada, P.; Ibarra, I. A.; Inge, A. K. A Robust and Biocompatible Bismuth Ellagate MOF Synthesized Under Green Ambient Conditions. *J. Am. Chem. Soc.* **2020**, *142* (39), 16795–16804. https://doi.org/10.1021/jacs.0c07525.

(2) P. H. M. Andrade, C. Volkringer, T. Loiseau, A. Tejeda, M. Hureau and A. Moissette, *Appl. Mater. Today*, 2024, 37, 102094.

(3) U. Noomnarm and R. M. Clegg, *Photosynth. Res.*, 2009, 101, 181–194.
